# A Comparison of Mercury Exposure from Seafood Consumption and Dental Amalgam Fillings in People with and without Amyotrophic Lateral Sclerosis (ALS): An International Online Case-Control Study

**DOI:** 10.3390/ijerph15122874

**Published:** 2018-12-14

**Authors:** Jane A. Parkin Kullmann, Roger Pamphlett

**Affiliations:** 1 The Stacey Motor Neuron Disease Laboratory, Discipline of Pathology, Brain and Mind Centre, Sydney Medical School, The University of Sydney, Sydney, NSW 2050, Australia; jpar5295@uni.sydney.edu.au; 2 Department of Neuropathology, Royal Prince Alfred Hospital, Sydney, NSW 2050, Australia

**Keywords:** amyotrophic lateral sclerosis, ALS, motor neuron disease, mercury, seafood, fish consumption, dental amalgam filling, case-control study, online questionnaire, international study

## Abstract

Exposures to toxic metals such as mercury have been suggested to be risk factors for amyotrophic lateral sclerosis (ALS). Human intake of mercury commonly occurs via consumption of seafood or from mercury-containing amalgam dental restorations (‘mercury fillings’). We therefore compared mercury exposures from these sources in 401 ALS and 452 non-ALS respondents, using an internationally-available online questionnaire that asked respondents how often they ate seafood and what their favourite types of seafoods were. Respondents were also asked to record numbers of current or former mercury fillings. ALS and non-ALS respondents did not differ in their frequency of seafood consumption or in monthly mercury intake from favourite seafoods. Both groups had similar numbers of current, as well as former, mercury fillings. In conclusion, this study found no evidence that mercury exposure from eating seafood, or from mercury dental fillings, was associated with the risk of developing ALS. Therefore, if mercury does play a role in the pathogenesis of ALS, other sources of exposure to mercury in the environment or workplace need to be considered. Alternatively, a susceptibility to mercury toxicity in ALS, such as genetic or epigenetic variations, multiple toxic metal interactions, or selenium deficiency, may be present.

## 1. Introduction

Toxic metals, and mercury in particular, have long been suspected to play a part in the pathogenesis of amyotrophic lateral sclerosis (ALS), also known as motor neuron disease (MND) [1,2,3]. Mercury can initiate oxygen free radical formation, induce excitotoxicity, reduce DNA, RNA and protein synthesis, cause epigenetic changes, activate autoimmunity, and interact with microtubules [4], all mechanisms that have been implicated in ALS [5]. Electron microscopic studies indicate that mercury binds selectively to intracellular sulfhydryl-rich membranes such as those of mitochondria, the nucleus, the Golgi apparatus, the endoplasmic reticulum and lysosomes [6], organelles whose functions have been reported to be impaired in ALS [5,7]. Systemically-administered mercury is taken up selectively by rodent spinal and brain stem motor neurons [8]. Mercury is also taken up preferentially by human spinal alpha motor neurons [9], spinal interneurons [10], and corticomotoneurons [11], and has been located in human astrocytes and oligodendrocytes [12]. All of these cells appear to play a part in the pathogenesis of ALS [5,13,14,15]. 

Environmental mercury remains a strong candidate as a precipitating factor for ALS, particularly if combined with a genetic predisposition to mercury toxicity [3]. However, despite toxic metals having biological plausibility and some epidemiological links with ALS, caution is advised regarding the limitations of methods used to assess human exposure [16]. One such limitation is that blood or cerebrospinal levels of toxic metals may not necessarily reflect the extent of previous exposure [17], since, for example, mercury remains in motor neurons for long periods after it has been removed from other organs [18]. Major sources of human exposure to mercury are via the consumption of seafood, especially of large predatory fish such as shark, swordfish, mackerel and tuna [19], and from mercury-containing ‘silver’ dental amalgam restorations [20], here termed ‘mercury fillings’ [21].

There is concern among people with ALS (as judged by online comments) as to whether they should attempt to reduce their mercury intake by having their dental amalgam fillings removed, or whether they should limit their fish intake. Interest has been rekindled in the mercury hypothesis for ALS [22] with a report of increased toenail mercury in people with ALS, possibly related to seafood consumption [23]. In addition, occasional reports have suggested that removing dental amalgam fillings, or chelation therapy, can result in stabilization or recovery from some forms of ALS [24]. We therefore sought to determine whether people with ALS are more likely than controls without ALS to be exposed to higher levels of mercury from these sources, using an online international questionnaire that gathered data on seafood and dental amalgam sources of mercury. Our results suggest that mercury exposure from these sources alone is not likely to be associated with the risk of developing ALS.

## 2. Methods

### 2.1. Setting

This case-control study used data collected between January 2015 and September 2017 from a multilingual web-based questionnaire, ALS Quest [25]. Cases were respondents who stated ‘Yes, I have been diagnosed with ALS/MND.’ Controls were participants who stated ‘No, I have not been diagnosed with ALS/MND.’ 

### 2.2. Ethics Approval 

The project was conducted in accordance with the Declaration of Helsinki, and was approved by the Human Ethics Committee of the Sydney Local Health District, reference number X14-0357. Responses used for the study were those where respondents consented by clicking an ‘I consent’ button and then submitted their responses. 

### 2.3. Frequency of Seafood Consumption

Participants were asked ‘How often do you eat fish or shellfish?’ on an increasing 8-point scale of: Never, Less than once a month, Once a month, 2–3 times a month, Once a week, 2–3 times a week, 4–6 times a week, or Daily. 

### 2.4. Favourite Seafoods

Seafood varies in its content of mercury, so participants were asked ‘Please list up to three of your favourite fish to eat’. Names of seafood entered in non-English languages were translated into English (unless it was a country-specific indigenous fish). The average mercury content of individual seafoods was based on the USA Food and Drug Administration (FDA) report of mercury levels in commercial fish and shellfish [19]. The mercury content of Australian seafood species not present in the USA FDA report was taken from an Australian report [26].

### 2.5. Estimation of Monthly Mercury Exposure from Seafood

**Figure 1:**
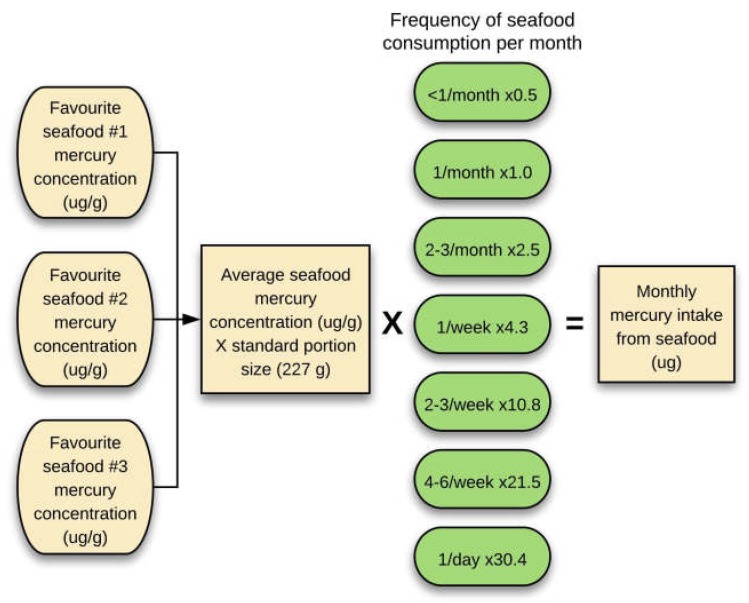
Estimating mercury exposure from seafood consumption. The average mercury concentration in µg/g from up to three favourite seafoods was multiplied by a standard portion size of seafood (227 g), then multiplied by the monthly frequency of seafood consumption to estimate monthly mercury exposure from seafood in micrograms.

The average mercury concentration in µg/g of up to three favourite seafoods was multiplied by 227 g (8 ounces, the weight of a typical seafood serving) [27], and then by the frequency of seafood consumption (adjusted to a monthly value), to get an estimate of monthly mercury exposure from seafood in micrograms (Figure 1). Assumptions underlying this estimate were that seafood mercury levels around the world are similar to those in the USA FDA list, and that the frequency of seafood consumption and favoured types of seafood remain reasonably stable over a long period of time. To compare types of seafood eaten, shellfish (e.g., prawn, crab and lobster) and cephalopods (e.g., squid, calamari and octopus) were categorised separately from finfish. 

### 2.6. Mercury-Containing Dental Fillings

Respondents were asked ‘Have you ever had an amalgam restoration (silver filling) as part of dental care?’. If they responded ‘Yes’ they were asked: ‘How many amalgam silver dental fillings do you currently have? For people with ALS/MND, enter the number you had before being diagnosed. You may need somebody to help you count the silver fillings in your mouth.’ Respondents were requested to indicate how many current fillings were occlusal, i.e., ‘those that involve the top surface of the tooth (where you bite)’ and non-occlusal, i.e., ‘those that involve the side of the tooth only’. If they had no current mercury fillings they were asked to enter ‘0’ for the current number of fillings, and then ‘If you currently have no silver amalgam dental fillings, how many have you had in the past?’ i.e., ‘former-only’ fillings. Responses were excluded if non-zero entries were made in both the current and former-only mercury filling categories, or if respondents entered ‘Yes’ to having ever had a filling but did not list any numbers of fillings. Respondents who responded ‘No’ were assigned a value of 0 for the number of current (combined occlusal and non-occlusal) and former-only fillings.

### 2.7. Statistical Analyses

Data from the Qualtrics server were transferred to IBM Statistical Package for the Social Sciences (SPSS) for Macintosh (version 22, IBM, Armonk, NY, USA) and GraphPad Prism 7 files. Extreme outliers (more than three times the inter-quartile range) in continuous variables were removed (numbers can be seen in the flow diagram). Nonparametric continuous variables were compared using Mann-Whitney U tests, and normally-distributed continuous variables with *t*-tests. Odds ratios with 95% confidence intervals and Fisher’s exact tests were used for categorical variables when all cell numbers were ≥5. Significance was assessed at the 0.05 level. No significant male vs. female differences were found in any of the seafood or mercury filling variables (data not shown, see Table S1) so the genders were analysed together.

## 3. Results

### 3.1. Cases and Controls

**Figure 2:**
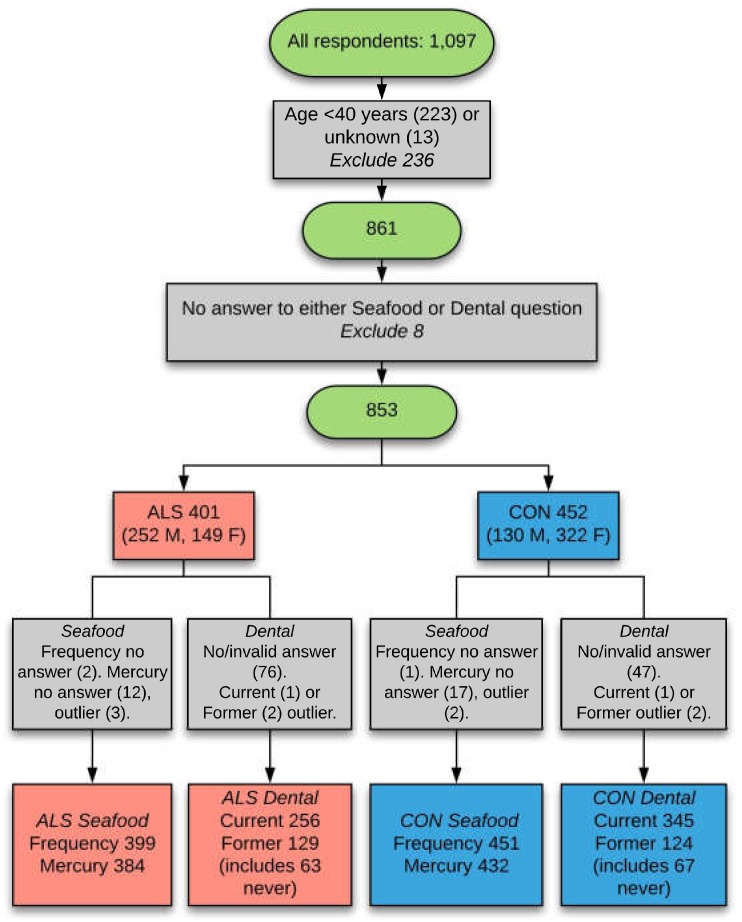
Selection of respondents for analysis. The final datasets of respondents used for analyses was achieved after exclusion criteria were applied for younger age, not answering questions on seafood consumption or dental fillings, and removal of outlier values. CON: control; current: current dental fillings; former: former-only dental fillings; frequency: frequency of seafood consumption; mercury: mercury content of favourite fish; never: replied ‘never had any current or former mercury fillings’; outlier: extreme outliers (more than three times the inter-quartile range); M: male; F: Female.

From an initial pool of 1097 questionnaire respondents, 853 eligible ones remained after inclusion criteria (i.e., aged 40 years and over, answered seafood and/or dental questions) were applied (Figure 2). 

These comprised 401 ALS respondents (252 male, 149 female) and 452 non-ALS controls (130 male, 322 female). The mean age of ALS respondents was 61.5 years (SD 9.2 years, range 40–87 years) and of controls was 57.3 years (SD 10.4 years, range 40–89 years), a significant difference on *t*-testing (*p* < 0.001). When evaluated by gender, the mean ages of male ALS respondents (62.0 years) and male controls (61.8 years) were not significantly different, while female ALS respondents (mean age 60.7 years) were older than female controls (mean age 55.5 years), *p* < 0.001 (see Table S2).

Common sources of information about the questionnaire cited by respondents were: ALS Associations (39%), the Internet (21%), friends (9%), ALS patients (6%), the USA Centers for Disease Control National ALS Registry (5%), health professionals (5%), community groups (4%), Facebook (4%), the Canadian Neuromuscular Disease Registry (2%) and ALS researchers (2%). The composition of the ALS and control groups was similar with regards to country of residence, ancestry and cultural group. The majority of respondents resided in Australia, the USA and Canada, though residents of a further 29 countries supplied responses (Table 1).

Eight percent of ALS respondents had at least one relative who had been diagnosed with ALS, and were considered to have familial ALS. The other 92% of ALS respondents were considered to have sporadic (or ‘isolated’) ALS. As reported by the ALS respondents, 58% had ‘classic’ (upper and lower motor neuron variant) ALS, 9% progressive muscular atrophy (lower motor neuron variant), 9% progressive bulbar palsy, 8% primary lateral sclerosis (upper motor variant), 8% ‘other’ and 8% did not know their subtype of ALS. Control respondents were friends (12%), spouses (11%), and blood or non-blood relatives (45%) of ALS patients, individuals from community, research or medical groups (9%), or other categories (22%).

The median online ALS Functional Rating Scale-Revised score [28] (inverted, so that higher scores indicated higher disability) was 13, and scores ranged from 0 to 48 (see Table S1). Most respondents were in the range of 6-18, with decreasing numbers as the scores increased, as expected in ALS [29]. The duration of ALS at the time of completing the questionnaire was calculated by subtracting the year of diagnosis from the year of consenting to complete the questionnaire. The median duration of disease was 1 year, with the great majority of respondents having disease durations of 4 years or fewer, as expected in ALS [29] (see Table S1).

### 3.2. Frequency of Seafood Consumption

**Figure 3:**
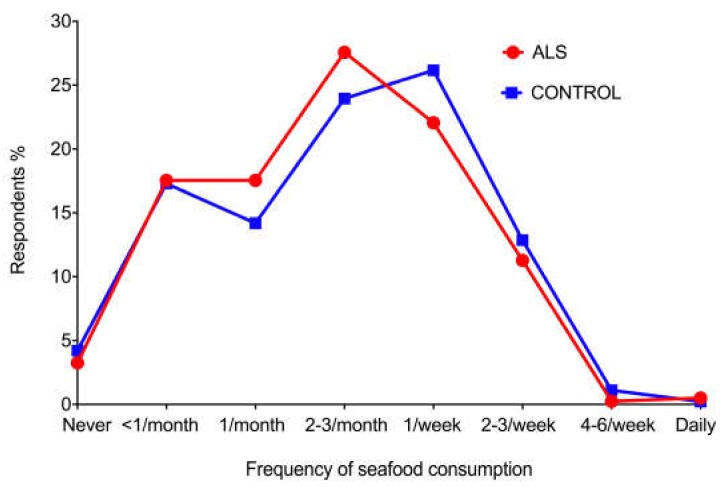
Proportion of respondents eating seafood at different frequencies. ALS and control respondents ate seafood at similar frequencies, ranging from never to daily. None of the slight differences in frequency proportions between ALS and controls was statistically significant.

A similar proportion of ALS (*N* = 386, 97%) and control (*N* = 432, 96%) respondents ate seafood. The proportions of respondents who ate seafood at different frequencies, ranging from never to daily, did not differ between ALS and control groups (Figure 3). Chi-square testing of ALS vs. control proportions at each of the eight frequencies showed no statistical differences (data not shown, see Table S1), even at frequencies where there appeared to be slight differences between ALS and controls, i.e., more for ALS at once per month and 2–3 per week, and more for controls at once per week. 

### 3.3. Favourite Seafoods

Of the 386 ALS respondents who said they ate seafood, 378 (98%) nominated at least one favourite type, while of the 432 control respondents who said they ate seafood, 417 nominated at least one favourite type (97%) (see Table S1). Some nominated favourite seafoods were not listed in the USA FDA or Australian seafood mercury reports; for the first choice these non-listed seafoods numbered 51 out of 796 cited (6%, 20 ALS and 31 controls), for the second choice 80 out of 737 cited (11%, 27 ALS and 53 controls), and for the third choice 69 out of 636 cited (11%, 27 ALS and 42 controls). A slightly greater proportion of ALS (*N* = 105, 28%) than control respondents (*N* = 84, 20%) cited one or more types of shellfish as a favourite type of seafood (OR = 1.5, 95% CI: 1.1–2.1, *p* = 0.01). No significant difference was found between the 5 ALS (1%) and 14 control (3%) respondents who cited cephalopods as a favourite seafood (OR = 0.4, 95% CI: 0.1–1.1, *p* = 0.07). 

### 3.4. Mercury Consumption from Favourite Seafoods

**Figure 4:**
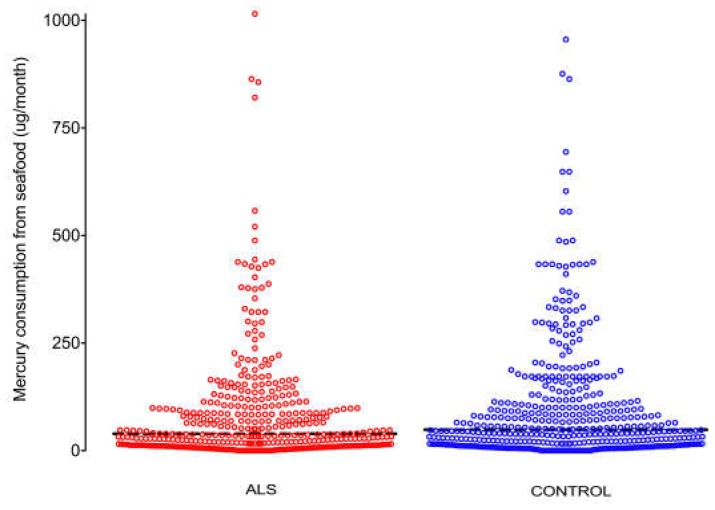
Distribution of mercury exposure from seafood. No difference is seen in the distribution of monthly mercury exposure from seafoods in micrograms between ALS and control respondents. Bar: median monthly exposure (µg).

No difference between ALS and control respondents was seen in the distribution of monthly mercury exposure from favourite seafoods (Figure 4). The monthly median value for seafood mercury exposure was slightly lower in ALS respondents (39 µg per month for ALS, 49 µg per month for controls) but these values did not differ significantly (*p* = 0.13) (see Table S1).

### 3.5. Intra- and Inter-Country Comparisons of Mercury Consumption from Favourite Seafoods

The two countries with the largest numbers of respondents were Australia and the USA, so monthly mercury consumption from seafood was compared between, and within, these countries, as well as with all other countries combined (Table 2, and see Table S1). Within all three nationality groups the amount of seafood mercury did not differ between ALS and control respondents. However, both Australian ALS and control respondents had higher seafood mercury consumption than corresponding USA groups. Further analysis showed this was because Australian respondents overall tended to eat seafoods with a higher mercury content (median 54 µg/month) than USA respondents (median 31 µg/month), while the frequency of seafood consumption was similar between these two countries (Australian median about 2–3 times per month and USA median slightly less than 2–3 times per month). For the other combined countries, seafood mercury intake (median 52 µg per month) was similar to that consumed by Australians, whereas the frequency of seafood consumption was higher (at slightly more than 2–3 times per month) than for Australia and the USA. 

### 3.6. Mercury-Containing Dental Fillings

**Figure 5:**
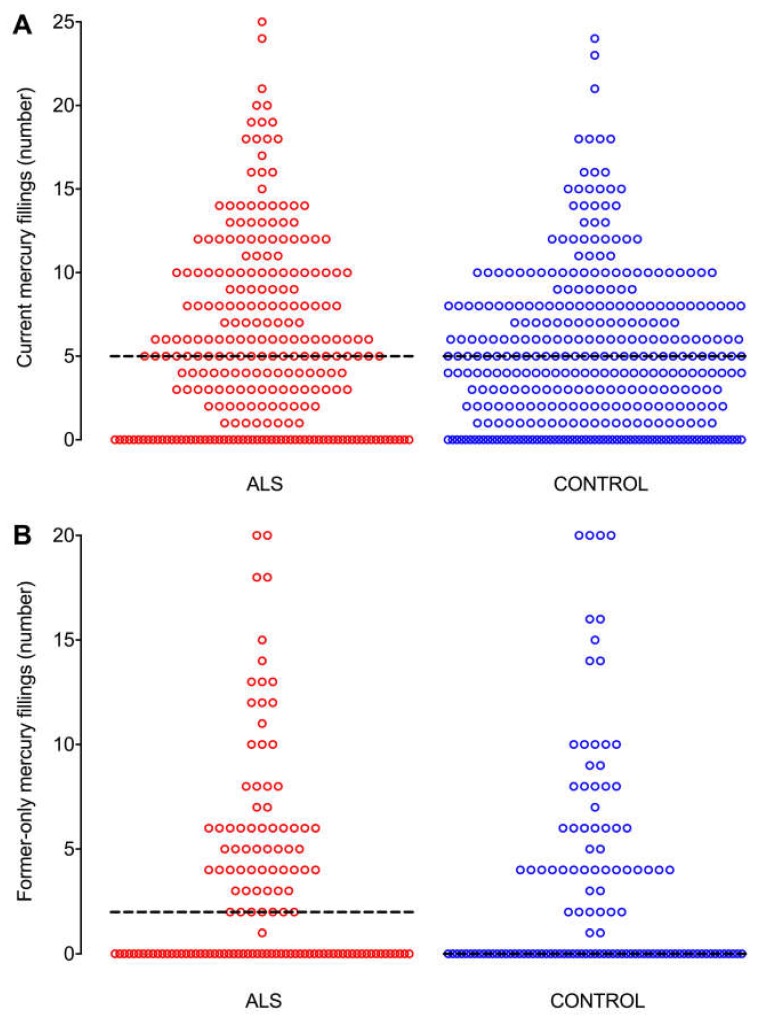
Distribution of numbers of current and former-only mercury fillings. The distribution of numbers of mercury fillings are similar between ALS and control groups for both (**A**) current fillings, and (**B**) former-only fillings. Bar: median number of fillings.

The proportions of ALS (*N* = 262, 81%) and control (*N* = 338, 83%) respondents who had ever had mercury fillings, i.e., either current (occlusal and non-occlusal) or former-only fillings, were similar (OR = 0.8, 95% CI: 0.6–1.2, *p* = 0.32), and see Table S1. The distribution of numbers of current fillings did not differ between ALS and control respondents (Figure 5A), with the median number of current fillings being 5 in both ALS and control groups (*p* = 0.25). ALS respondents (*N* = 188, 58%) were less likely than controls (*N* = 264, 65%) to have current occlusal fillings (OR = 0.7, 95% CI: 0.5–1.0, *p* = 0.04). The distribution of numbers of former-only fillings was similar between ALS and control groups (Figure 5B), with medians of 2 former-only fillings in ALS respondents and 0 in controls (*p* = 0.46).

### 3.7. Intra- and Inter-Country Comparisons of Mercury-Containing Dental Fillings

No differences were seen in median numbers of current (occlusal and non-occlusal) mercury fillings, either between ALS and control respondents who resided within each of the USA, Australia, and other countries combined, or between USA and Australian ALS and control respondents (Table 3, and see Table S1).

## 4. Discussion

Key findings in this study are that people with and without ALS had comparable intakes of mercury from their favourite seafoods, and had similar numbers of current or former mercury fillings. In fact, ALS respondents were *less* likely than controls to have occlusal mercury fillings, which are more likely to be associated with higher levels of intra-oral air mercury because of chewing [30]. Our study therefore found no convincing evidence that people with ALS are exposed to more environmental mercury than controls from either seafood consumption or from mercury-containing dental fillings.

The U.S. Environmental Protection Agency’s acceptable daily dose for methylmercury, i.e., the dose that would not be anticipated to result in any adverse health effects, is 0.1 µg/kg per day [31]. Multiplied by 80 kg (the weight of a typical adult) and 30.8 (the average number of days in a month), this gives a value of 246 µg of mercury per month. The median monthly seafood mercury consumption values for our respondents, 39 µg/month for ALS and 49 µg/month for controls, are well below what the U.S. Environmental Protection Agency considers the maximum acceptable level of exposure for methylmercury, assuming that all the mercury in seafood is present as methylmercury. Therefore, the seafood-related mercury consumption we found in most of our respondents would not be expected to be toxic in the absence of complicating susceptibility factors.

Shellfish, which were more likely to be consumed by our ALS respondents, are low in mercury (0.003–0.100 µg/g) but have been found to contain beta-N-methylamino-L-alanine (BMAA) [32], a postulated risk factor for ALS [33]. This finding therefore supports further research into links between seafood consumption, BMAA and ALS.

The failure to find an increase in mercury exposure from common environmental sources in people with ALS raises the possibility that some susceptibility factors could be responsible for activating mercury that can lie dormant within human motor neurons [9,10,11]. This susceptibility could be due to genetic polymorphisms, since a number of genetic variants are implicated in susceptibility to mercury toxicity [34], or to acquired epigenetic differences [35,36], both in accordance with the notion that environmental insults, combined with genetic susceptibility and ageing, trigger the common sporadic form of ALS [37]. Of interest in this regard, a gene-environment interaction involving mercury and mutant superoxide dismutase 1 has been described, which results in increased calcium-mediated glutamate excitotoxicity [38]. Mercury is found often in the human nervous system, since almost half of subjects in an autopsy population from different clinicopathological backgrounds have detectable mercury in the brain stem locus ceruleus, a site that appears to be a marker for previous exposure to mercury [39]. These findings raise the possibility that mercury could be one of the non-genetic factors in a multistep process that is thought to underlie ALS [40]. Susceptibility to mercury in ALS could also arise from multiple toxic metal interactions [41], or from selenium deficiency, since selenium mitigates mercury toxicity [42] and low selenium levels have been implicated in a number of neurodegenerative diseases [43].

Our findings provide no evidence that mercury exposure from eating seafood or from dental fillings is related to ALS, but there remain other sources of mercury exposure that were not assessed in this study. For example, mortality rates from ALS have been found to higher in municipalities of Spain where levels above regulatory limits of heavy metals such as mercury are released into rivers [44]. Mercury is also used in many industrial applications, such as the production of chlorine gas and caustic soda, and is also present in thermometers, barometers, batteries, and electrical switches. If appropriate workplace protections are not in place in these occupational settings, exposure to mercury could occur [45]. Some occupational studies give indirect evidence that workplace exposure to mercury in factories may be implicated in ALS because men with ALS tend to have occupations involving lower skills and tasks, typically associated with factory workers [46]. A recent systematic review of occupational exposures in ALS lists 15 studies in which occupational exposure to toxic metals was reported [47]. In five of these, mercury was analysed as an individual metal [48,49,50,51,52], but case and control numbers were too small in all of these reports for robust statistical analyses.

Another factor to consider, before rejecting the notion of exposure to mercury being related to ALS, is that ocean levels of mercury are less than a quarter of those expected from known mercury emissions such as coal burning, cement production, waste incineration and small-scale gold mining [53]. This has led to the concept of ‘missing mercury’ [54] and implies that people may be exposed to mercury without ever being aware of its source. These other sources of mercury exposure are not able to be identified using epidemiological methods, so an alternative approach is to look directly for mercury in the tissues of people with ALS. These can be either peripheral tissues, such as toenails [23], or central nervous system tissues where mercury, together with other toxic metals, can now be localised within individual regions and cells using methods such as autometallography [9,10,11,55] and laser ablation-inductively coupled plasma-mass spectrometry [39]. 

Of interest is the finding that people in the USA eat seafood with a lower mercury content than do people in Australia (and other combined countries), whereas the frequency of seafood consumption in these two countries is similar. This did not affect our finding of no increased mercury consumption from eating seafood in ALS respondents, but it may have implications for epidemiological studies of mercury-associated disorders when comparisons are made between countries. This finding shows the value of internationally-available surveys in looking at disorders that could be triggered by environmental agents.

The limitations of online questionnaires in the investigation of ALS risk factors have recently been summarised [56]. Limitations specific to the present study are: (1) The female ALS respondents were older than their female controls. However, this means that the ALS females would have had a longer period of time in which to accumulate environmental mercury from the two sources of exposure, but no female ALS-control exposure differences were found to indicate this was the case (data not shown). (2) We were not able to trace the mercury content of a small number of reported favourite seafoods, but the proportion of these was similar in ALS and control respondents. (3) Recall bias is always a concern in case-control studies, but ALS and control respondents gave similar depths of detail in response to questions on seafood consumption and dental fillings, suggesting that recall bias is unlikely to play a major in this study. Furthermore, since we found no differences between ALS and control respondents, any recall bias on the part of control respondents (who could tend to be less assiduous in recalling seafood consumption or numbers of dental fillings) implies that control respondents actually consumed more seafood and had more amalgam filling than they reported, which would reinforce our finding that these exposures are not risk factors for ALS.

## 5. Conclusions

This international online study did not find evidence that mercury exposure from either seafood consumption or from mercury-containing dental fillings is more common in people with ALS than in controls. If mercury does play a role in the pathogenesis of ALS, it seems likely that other factors such as genetic or epigenetic susceptibilities to mercury toxicity, multiple toxic metal interactions, or selenium deficiency would need to be present to trigger the disease, or that a link between mercury and ALS could arise from other as yet undefined environmental or workplace sources. Further international comparisons of mercury intake in ALS from fish consumption and dental amalgam fillings will be possible once we obtain more responses to our ongoing online risk factor questionnaire.

## Figures and Tables

**Table 1:** Demographic characteristics of respondents.

	ALS	*N* (%)	Control	*N* (%)
Country of residence				
	United States	180 (45%)	Australia	339 (75%)
	Australia	116 (29%)	United States	49 (11%)
	Canada	57 (14%)	Other * (<2% each)	36 (8%)
	Other * (<2% each)	37 (9%)	Spain	14 (3%)
	Spain	9 (2%)	New Zealand	12 (3%)
Ancestry				
	Other (<6% each)	184 (47%)	Other (<4% each)	154 (34%)
	Australian	60 (15%)	Australian	131 (29%)
	English	57 (15%)	English	77 (17%)
	American	37 (9%)	Irish	41 (9%)
	German	32 (8%)	British	25 (6%)
	Irish	23 (6%)	Scottish	19 (4%)
Cultural group				
	American	112 (29%)	Australian	286 (64%)
	Australian	98 (26%)	Other (<2% each)	76 (17%)
	Other (<3% each)	93 (24%)	American	33 (7%)
	Canadian	40 (10%)	English	27 (6%)
	English	28 (7%)	Spanish	14 (3%)
	German	13 (3%)	New Zealander	11 (2%)

Other * (countries of residence): Argentina, Belgium, Brazil, Cape Verde, China, Colombia, Czech Republic, Denmark, Ecuador, Egypt, Finland, Germany, Iran, Ireland, Italy, Luxembourg, Mexico, Netherlands, Portugal, Russia, Slovakia, South Africa, South Korea, Sweden, Switzerland, Turkey, United Kingdom (country not specified).

**Table 2:** Intra- and inter-country comparisons of monthly mercury consumption from seafood.

	Respondents *N*	Median Mercury Consumption µg/month	*p*	r
Intra-country: Control vs. ALS				
USA control	47	32	0.80	0.02
USA ALS	177	31		
Australia control	324	51	0.27	0.05
Australia ALS	109	69		
Other countries control	61	65	0.40	0.07
Other countries ALS	98	47		
Inter-country: USA vs. Australia				
USA control	47	32	0.02	0.12
Australia control	324	51		
USA ALS	177	31	<0.001	0.21
Australia ALS	109	69		

*N*: number, *p*: Mann-Whitney *p* value, r: effect size.

**Table 3:** Intra- and inter-country comparisons of current mercury amalgam dental fillings.

	Respondents *N*	Median No. of Current Fillings	*p*	r
Intra-country: Control vs. ALS				
USA control	30	4.5	0.49	0.06
USA ALS	103	5.0		
Australia control	270	5.0	0.42	0.04
Australia ALS	84	4.5		
Other countries control	45	5.0	0.76	0.03
Other countries ALS	69	5.0		
Inter-country: USA vs. Australia				
USA control	30	4.5	0.93	0.005
Australia control	270	5.0		
USA ALS	103	5.0	0.80	0.02
Australia ALS	84	4.5		

*N*: number, *p*: Mann-Whitney *p* value, r: effect size.

## Supplementary Materials

The following are available online at http://www.mdpi.com/1660-4601/15/12/2874/s1, Table S1 Seafood and dental filling data, Table S2. Ages of respondents.

## References

[1] Brown I.A. (1954). Chronic mercurialism: A cause of the clinical syndrome of amyotrophic lateral sclerosis. AMA Arch. Neurol. Psychiatry.

[2] Adams C.R., Ziegler D.K., Lin J.T. (1983). Mercury intoxication simulating amyotrophic lateral sclerosis. JAMA.

[3] Johnson F.O., Atchison W.D. (2009). The role of environmental mercury, lead and pesticide exposure in development of amyotrophic lateral sclerosis. Neurotoxicology.

[4] Bjorklund G., Dadar M., Mutter J., Aaseth J. (2017). The toxicology of mercury: Current research and emerging trends. Environ. Res..

[5] Hardiman O., Al-Chalabi A., Chio A., Corr E.M., Logroscino G., Robberecht W., Shaw P.J., Simmons Z., van den Berg L.H. (2017). Amyotrophic lateral sclerosis. Nat. Rev. Dis. Primers.

[6] Chang L.W., Hartmann H.A. (1972). Electron microscopic histochemical study on the localization and distribution of mercury in the nervous system after mercury intoxication. Exp. Neurol..

[7] Haase G., Rabouille C. (2015). Golgi Fragmentation in ALS Motor Neurons. New Mechanisms Targeting Microtubules, Tethers, and Transport Vesicles. Front. Neurosci..

[8] Arvidson B. (1992). Inorganic mercury is transported from muscular nerve terminals to spinal and brainstem motoneurons. Muscle Nerve.

[9] Pamphlett R., Waley P. (1998). Mercury in human spinal motor neurons. Acta Neuropathol..

[10] Pamphlett R., Kum Jew S. (2016). Age-Related Uptake of Heavy Metals in Human Spinal Interneurons. PLoS ONE.

[11] Pamphlett R., Kum Jew S. (2013). Uptake of inorganic mercury by human locus ceruleus and corticomotor neurons: Implications for amyotrophic lateral sclerosis. Acta Neuropathol. Commun..

[12] Pamphlett R., Kum Jew S. (2018). Inorganic mercury in human astrocytes, oligodendrocytes, corticomotoneurons and the locus ceruleus: Implications for multiple sclerosis, neurodegenerative disorders and gliomas. Biometals.

[13] Eisen A., Kim S., Pant B. (1992). Amyotrophic lateral sclerosis (ALS): A phylogenetic disease of the corticomotoneuron?. Muscle Nerve.

[14] Turner M.R., Kiernan M.C. (2012). Does interneuronal dysfunction contribute to neurodegeneration in amyotrophic lateral sclerosis?. Amyotroph. Lateral Scler..

[15] Philips T., Bento-Abreu A., Nonneman A., Haeck W., Staats K., Geelen V., Hersmus N., Kusters B., Van Den Bosch L., Van Damme P. (2013). Oligodendrocyte dysfunction in the pathogenesis of amyotrophic lateral sclerosis. Brain.

[16] Vinceti M., Bottecchi I., Fan A., Finkelstein Y., Mandrioli J. (2012). Are environmental exposures to selenium, heavy metals, and pesticides risk factors for amyotrophic lateral sclerosis?. Rev. Environ. Health.

[17] Vinceti M., Filippini T., Mandrioli J., Violi F., Bargellini A., Weuve J., Fini N., Grill P., Michalke B. (2017). Lead, cadmium and mercury in cerebrospinal fluid and risk of amyotrophic lateral sclerosis: A case-control study. J. Trace Elem. Med. Biol..

[18] Pamphlett R., Waley P. (1996). Motor neuron uptake of low dose inorganic mercury. J. Neurol. Sci..

[19] Mercury Levels in Commercial Fish and Shellfish (1990–2012). https://www.fda.gov/food/foodborneillnesscontaminants/metals/ucm115644.htm.

[20] About Dental Amalgam Fillings. https://www.fda.gov/medicaldevices/productsandmedicalprocedures/dentalproducts/dentalamalgam/ucm171094.htm.

[21] Clarkson T.W., Magos L. (2006). The toxicology of mercury and its chemical compounds. Crit. Rev. Toxicol..

[22] Ho D.T., Russell J.A. Mercury and motor neuron disease: Hooked on a hypothesis. Muscle Nerve.

[23] Andrew A.S., Chen C.Y., Caller T.A., Tandan R., Henegan P.L., Jackson B.P., Hall B.P., Bradley W.G., Stommel E.W. Toenail mercury levels are associated with amyotrophic lateral sclerosis risk. Muscle Nerve.

[24] Mangelsdorf I., Walach H., Mutter J. (2017). Healing of Amyotrophic Lateral Sclerosis: A Case Report. Complement. Med. Res..

[25] Parkin Kullmann J.A., Hayes S., Wang M.X., Pamphlett R. (2015). Designing an Internationally Accessible Web-Based Questionnaire to Discover Risk Factors for Amyotrophic Lateral Sclerosis: A Case-Control Study. JMIR Res. Protoc..

[26] Padula D., Greenfield H., Cunningham J., Kiermeier A., McLeod C. (2016). Australian seafood compositional profiles: A pilot study. Vitamin D and mercury content. Food Chem..

[27] Guidance for Assessing Chemical Contaminant Data for Use in Fish Advisories, Volume 2, Risk Assessment and Fish Consumption Limits 2000. https://www.epa.gov/quality/guidance-assessing-chemical-contaminant-data-use-fish-advisories-volume-2-risk-assessment.

[28] Maier A., Holm T., Wicks P., Steinfurth L., Linke P., Munch C., Meyer R., Meyer T. (2012). Online assessment of ALS functional rating scale compares well to in-clinic evaluation: A prospective trial. Amyotroph. Lateral Scler..

[29] Chio A. (1999). ISIS Survey: An international study on the diagnostic process and its implications in amyotrophic lateral sclerosis. J. Neurol..

[30] Vimy M.J., Lorscheider F.L. (1985). Serial measurements of intra-oral air mercury: Estimation of daily dose from dental amalgam. J. Dent. Res..

[31] Methylmercury (MeHg). https://cfpub.epa.gov/ncea/iris2/chemicallanding.cfm?substance_nmbr=73.

[32] Banack S.A., Metcalf J.S., Bradley W.G., Cox P.A. (2014). Detection of cyanobacterial neurotoxin beta-N-methylamino-l-alanine within shellfish in the diet of an ALS patient in Florida. Toxicon.

[33] Caller T.A., Field N.C., Chipman J.W., Shi X., Harris B.T., Stommel E.W. (2012). Spatial clustering of amyotrophic lateral sclerosis and the potential role of BMAA. Amyotroph. Lateral Scler..

[34] Andreoli V., Sprovieri F. (2017). Genetic Aspects of Susceptibility to Mercury Toxicity: An Overview. Int. J. Environ. Res. Public Health.

[35] Basu N., Goodrich J.M., Head J. (2014). Ecogenetics of mercury: From genetic polymorphisms and epigenetics to risk assessment and decision-making. Environ. Toxicol. Chem..

[36] Young P.E., Kum Jew S., Buckland M.E., Pamphlett R., Suter C.M. (2017). Epigenetic differences between monozygotic twins discordant for amyotrophic lateral sclerosis (ALS) provide clues to disease pathogenesis. PLoS ONE.

[37] Yu B., Pamphlett R. (2017). Environmental insults: Critical triggers for amyotrophic lateral sclerosis. Transl. Neurodegener..

[38] Bailey J.M., Colon-Rodriguez A., Atchison W.D. (2017). Evaluating a Gene-Environment Interaction in Amyotrophic Lateral Sclerosis: Methylmercury Exposure and Mutated SOD1. Curr. Environ. Health Rep..

[39] Pamphlett R., Bishop D.P., Kum Jew S., Doble P.A. (2018). Age-related accumulation of toxic metals in the human locus ceruleus. PLoS ONE.

[40] Chio A., Mazzini L., D’Alfonso S., Corrado L., Canosa A., Moglia C., Manera U., Bersano E., Brunetti M., Barberis M. (2018). The multistep hypothesis of ALS revisited: The role of genetic mutations. Neurology.

[41] Andrade V.M., Aschner M., Marreilha Dos Santos A.P. (2017). Neurotoxicity of Metal Mixtures. Adv. Neurobiol..

[42] Bjorklund G., Aaseth J., Ajsuvakova O.P., Nikonorov A.A., Skalny A.V., Skalnaya M.G., Tinkov A.A. (2017). Molecular interaction between mercury and selenium in neurotoxicity. Coordin. Chem. Rev..

[43] Cardoso B.R., Roberts B.R., Bush A.I., Hare D.J. (2015). Selenium, selenoproteins and neurodegenerative diseases. Metallomics.

[44] Sanchez-Diaz G., Escobar F., Badland H., Arias-Merino G., Posada de la Paz M., Alonso-Ferreira V. (2018). Geographic Analysis of Motor Neuron Disease Mortality and Heavy Metals Released to Rivers in Spain. Int. J. Environ. Res. Public Health.

[45] Mercury. https://www.cdc.gov/niosh/topics/mercury/default.html.

[46] Pamphlett R., Rikard-Bell A. (2013). Different occupations associated with amyotrophic lateral sclerosis: Is diesel exhaust the link?. PLoS ONE.

[47] Gunnarsson L.G., Bodin L. (2018). Amyotrophic Lateral Sclerosis and Occupational Exposures: A Systematic Literature Review and Meta-Analyses. Int. J. Environ. Res. Public Health.

[48] Gresham L.S., Molgaard C.A., Golbeck A.L., Smith R. (1986). Amyotrophic lateral sclerosis and occupational heavy metal exposure: A case-control study. Neuroepidemiology.

[49] Gunnarsson L.G., Bodin L., Soderfeldt B., Axelson O. (1992). A case-control study of motor neurone disease: Its relation to heritability, and occupational exposures, particularly to solvents. Br. J. Ind. Med..

[50] McGuire V., Longstreth W.T., Nelson L.M., Koepsell T.D., Checkoway H., Morgan M.S., van Belle G. (1997). Occupational exposures and amyotrophic lateral sclerosis. A population-based case-control study. Am. J. Epidemiol..

[51] Yu Y., Su F.C., Callaghan B.C., Goutman S.A., Batterman S.A., Feldman E.L. (2014). Environmental risk factors and amyotrophic lateral sclerosis (ALS): A case-control study of ALS in Michigan. PLoS ONE.

[52] Andrew A.S., Caller T.A., Tandan R., Duell E.J., Henegan P.L., Field N.C., Bradley W.G., Stommel E.W. (2017). Environmental and Occupational Exposures and Amyotrophic Lateral Sclerosis in New England. Neurodegener. Dis..

[53] Lamborg C.H., Hammerschmidt C.R., Bowman K.L., Swarr G.J., Munson K.M., Ohnemus D.C., Lam P.J., Heimburger L.E., Rijkenberg M.J., Saito M.A. (2014). A global ocean inventory of anthropogenic mercury based on water column measurements. Nature.

[54] Missing Mercury Pollution Is Enough for Mass Poisoning. https://www.newscientist.com/article/mg22329811-800-missing-mercury-pollution-is-enough-for-mass-poisoning/.

[55] Pamphlett R., Jew S.K. (2013). Heavy metals in locus ceruleus and motor neurons in motor neuron disease. Acta Neuropathol. Commun..

[56] Parkin Kullmann J.A., Pamphlett R. (2017). Does the index-to-ring finger length ratio (2D:4D) differ in amyotrophic lateral sclerosis (ALS)? Results from an international online case-control study. BMJ Open.

